# Ultrasound for the Diagnosis of Biliary Atresia: From Conventional Ultrasound to Artificial Intelligence

**DOI:** 10.3390/diagnostics12010051

**Published:** 2021-12-27

**Authors:** Wenying Zhou, Luyao Zhou

**Affiliations:** Department of Medical Ultrasonics, Institute for Diagnostic and Interventional Ultrasound, The First Affiliated Hospital, Sun Yat-sen University, No 58, Zhongshan Er Road, Guangzhou 510080, China; zhouwy6@mail2.sysu.edu.cn

**Keywords:** biliary atresia, imaging, conventional ultrasound, elastography, percutaneous cholecystocholangiography, artificial intelligence

## Abstract

Biliary atresia is an aggressive liver disease of infancy and can cause death without timely surgical intervention. Early diagnosis of biliary atresia is critical to the recovery of bile drainage and long-term transplant-free survival. Ultrasound is recommended as the initial imaging strategy for the diagnosis of biliary atresia. Numerous ultrasound features have been proved helpful for the diagnosis of biliary atresia. In recent years, with the help of new technologies such as elastography ultrasound, contrast-enhanced ultrasound and artificial intelligence, the diagnostic performance of ultrasound has been significantly improved. In this review, various ultrasound features in the diagnosis of biliary atresia are summarized. A diagnostic decision flow chart for biliary atresia is proposed on the basis of the hybrid technologies, combining conventional ultrasound, elastography and contrast-enhanced ultrasound. In addition, the application of artificial intelligence in the diagnosis of biliary atresia with ultrasound images is also introduced.

## 1. Introduction

Biliary atresia (BA) is a severe obliterative fibrosing cholangiopathy of infancy, with a worldwide prevalence ranging from 1 in 5000 to 19,000 livebirths [1,2,3]. If left untreated, BA can lead to progressive liver cirrhosis, and finally result in death from hepatic failure by age 2 years [4]. Kasai portoenterostomy (KPE) is the primary treatment to restore bile flow for patients with BA. The prognosis of KPE for patients less than 60 days is significantly better than that for those more than 60 days [5]. Liver transplantation is consequently performed if KPE fails to restore bile flow or other severe liver-related events occur. Thus, early diagnosis of BA is extremely critical, as early surgical intervention is required to achieve long-term transplant-free survival.

The clinical symptoms of infants with BA mainly include persistent jaundice, pale stools and dark urine shortly after birth [4]. Such clinical symptoms are caused by cholestasis, which is characterized of conjugated hyperbilirubinemia (conjugated bilirubin level of ≥2 mg/dL and >20% of the total bilirubin) [4,6]. However, the above clinical symptoms usually overlap with other causes of neonatal cholestasis in the first few weeks of life. For the early diagnosis of BA, researchers have endeavored to screen the direct bilirubin concentration in the first week of life [7,8] or stool color [2,9] in newborns, which yielded the sensitivities of 97.1–100% [2,7,8,9]. Furthermore, serum gamma-glutamyl transferase (GGT) is usually higher in BA than in other causes of neonatal cholestasis [4,10]. A markedly elevated GGT level (>150–200 U/l) usually suggests BA [10]. Therefore, those infants with elevated conjugated bilirubin level and GGT levels beyond 2 weeks of age should be alerted to the possibility of BA and prompted further investigation.

Infants with suspected BA need to undergo imaging examinations for further evaluation of the potential anomaly of biliary system. There are several modalities widely chosen for the evaluation including ultrasonography (US), hepatobiliary scintigraphy and magnetic resonance cholangiography (MRCP). Hepatobiliary scintigraphy can be used for the assessment of the patency of the bile duct by observing the excretion of the tracer into the small intestine, but the specificity was as low as 70.4% [11]. Furthermore, it is time-consuming and radiation-active [12]. MRCP permits the evaluation of the existence of an extrahepatic bile duct, but the need for sedation and the insufficient spatial resolution due to small body size limit it from being the preferred strategy [13,14].

Ultrasonography (US), which is a radiation-free, noninvasive and real time imaging modality, has been recommended as the preferred imaging tool for the initial detection of BA [4,11]. Multi-modal US technology has been applied to the diagnosis of BA. In this review, a variety of US techniques including conventional ultrasound, elastography ultrasound and contrast-enhanced ultrasound in the diagnosis of BA will be outlined. In addition, the application of artificial intelligence (AI) in the diagnosis of BA with US images is also introduced.

## 2. Conventional Ultrasound

Conventional US is comprised of grey scale US and color Doppler US. With the help of constantly updated US equipment, the US features can be observed clearly better than ever before. High-frequency ultrasound (>10 MHz) is suggested for grey scale US and color Doppler to achieve the best spatial resolution. Numerous US features, especially the combination of gallbladder abnormalities and the triangular cord (TC) sign [11], have been proved helpful for diagnosing BA. Either of the two is positive, the infant should receive surgical exploration or cholangiography for the exclusion of BA. Other features have also been reported. The details are as follows.

### 2.1. Gallbladder Abnormalities

Gallbladder abnormality is the earliest and most widely used US feature for diagnosing BA, usually with both sensitivity and specificity more than 90% [11,15,16]. On US, a normal gallbladder is displayed with a complete and smooth hyperechoic mucosal lining, regardless of whether the gallbladder lumen is completely filled or not. Due to congenital dysplasia of the gallbladder, the size or the morphology of the gallbladder of infant with BA is usually abnormal. The definitions of gallbladder abnormalities have varied among different studies [15,16,17,18,19,20]. The length of the gallbladder, the integrity of the mucosal lining, and the degree of contraction of the gallbladder after feeding had all been reported to identify BA [15,16,17,18,19,20].

**Figure 1:**
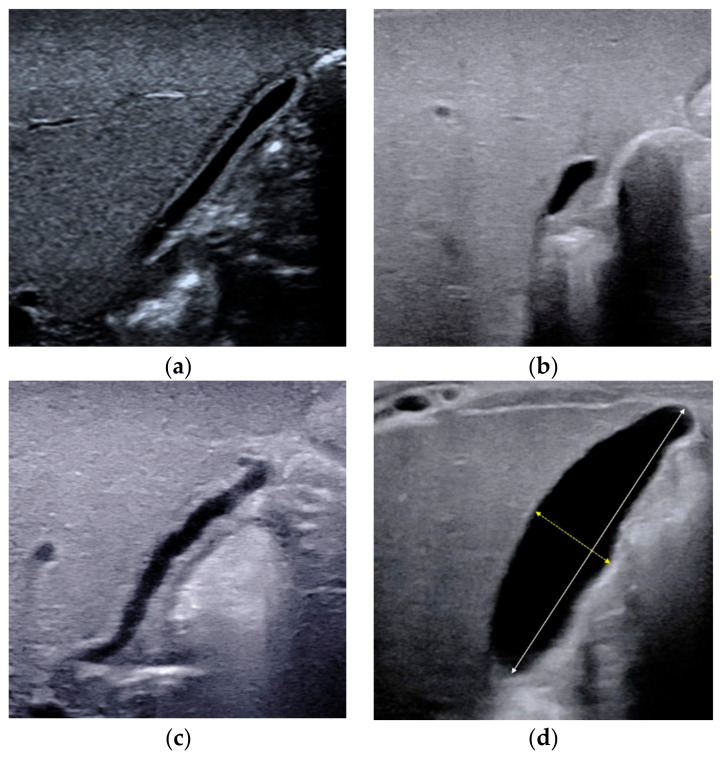
Different types of gallbladders detected by conventional ultrasound. (**a**) Type II gallbladder in non-BA. (**b**) Type III gallbladder in BA. (**c**) Type IV gallbladder with length-to-width ratios of >5.2 in BA. (**d**) Type IV gallbladder with length-to-width ratios of ≤5.2 in non-BA. The maximum lumen length and width should be measured from inner wall to inner wall.

Combining the experience of our center and the results of previous studies, a gallbladder classification scheme was proposed in 2015 for the diagnosis of BA [20], including four types of gallbladders: Type I, dysplastic gallbladder, in which the gallbladder was not detected; Type II, unfilled gallbladder, in which a gallbladder was detected with incompletely filled lumen and with smooth and complete hyperechogenic mucosal lining (Figure 1a); Type III, small gallbladder, in which a gallbladder was detected with a fully filled lumen and the length of the lumen <1.5 cm (Figure 1b); Type IV, in which a gallbladder was detected with a fully filled lumen and the length of the lumen more than 1.5 cm (Figure 1c,d). For type IV, the maximum length and width of the gallbladder were measured from inner wall to inner wall (Figure 1d), and the length-to-width ratio was calculated. In particular, when measuring the length of the gallbladder, it is necessary to perform a segmental measurement according to the meandering of the gallbladder. An abnormal gallbladder was defined as type I, type III, and type IV with length-to-width ratios of >5.2 (Figure 1c), and used to predict BA. A normal gallbladder was defined as type II and type IV gallbladders with length-to-width ratios of ≤5.2 (Figure 1d), and used to predict non-BA. This classification scheme yielded a sensitivity of 86.8% and a specificity of 90.3% [20]. It was proved that this scheme could yield similar diagnostic performance even if the infant is fasted for less than 4 h [21]. No enlargement or contraction of the gallbladder after feeding was reported as another useful indicator for the diagnosis of BA [18]. However, the contraction of the gallbladder after feeding can’t rule out BA.

Although the gallbladder abnormalities yield both high sensitivity and high specificity in the diagnosis of BA, there are still some infants with BA that have normal gallbladder. In these cases, other US features, such as TC sign and porta hepatis cyst, are required with further investigation to prevent them from being missed.

### 2.2. Triangular Cord Sign

**Figure 2:**
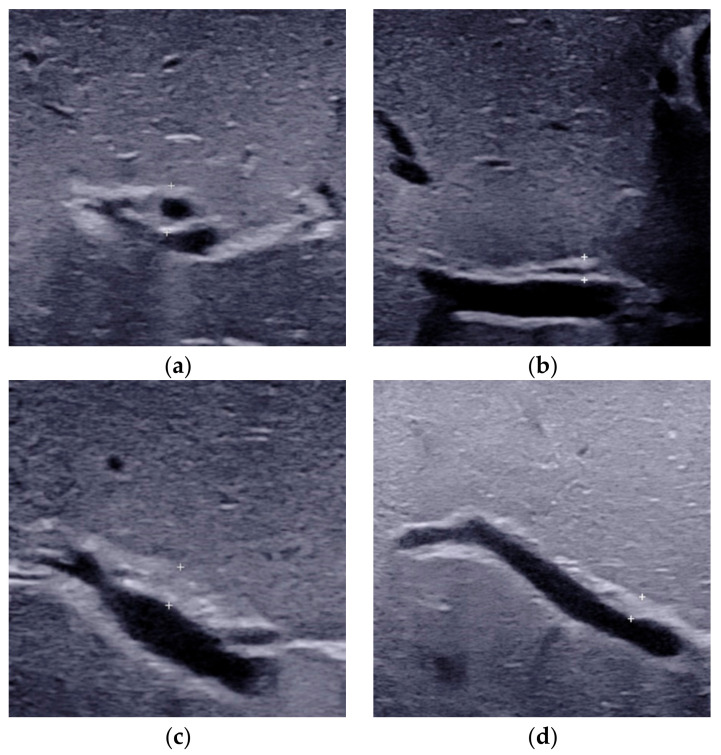
The triangular cord thickness measured above the anterior branch of the right portal vein on a longitudinal image. (**a**) The TC thickness >4.0mm including HA in BA. (**b**) The TC thickness <4.0 mm including HA in non-BA. (**c**) The TC thickness >2.0 mm not including HA in BA. (**d**) The TC thickness <2.0 mm not including HA in non-BA. TC, triangular cord; HA, hepatic artery; BA, biliary atresia.

The TC thickness is very sensitive in the identification of BA [20,22,23,24]. It was firstly defined as the triangular or tubular-shaped echogenic density above the portal vein on a transverse or longitudinal image by Choi et al. [23] in 1996. Subsequent studies had also proved that TC sign was a helpful indicator for diagnosing BA [11,22,24]. However, the location and the cut-off value of TC thickness in different studies varied. The most frequently used TC sign is defined as the echogenic area >4 mm including hepatic artery (HA) (Figure 2a), located at the right portal vein on a longitudinal scan [22]. Patients with a thickness value less than 4 mm (Figure 2b) were deemed as without BA. As there is no consensus in the literature on how or where to measure before, resulting in individual differences in visualization and measurement, with reported sensitivities varying from 23 to 100% [11,25,26].

To overcome the shortcomings of the already existed TC thickness, we proposed a modified TC thickness, which was defined as the thickness of the echogenic anterior wall of the anterior branch of the right portal vein just distal to the right portal vein on a longitudinal image (Figure 2c), without including the right HA [20]. The modified TC thickness >2 mm was considered as a positive sign to predict BA. On the other hand, patients with a thickness value less than 2 mm (Figure 2d) were deemed as without BA. The diagnostic performance of this modified TC sign could be comparable or superior to that of the gallbladder abnormalities, with the areas under the receiver operating characteristic curve (AUC) ranged from 0.771 to 0.952 [20,21,27,28]. Higher AUC could be obtained when modified TC thickness combined with the gallbladder abnormalities [20,21,27,28]. Furthermore, the combination of the modified TC sign, gallbladder classification scheme and GGT level with a cut off-value of 188 IU/L could yielded the sensitivity of 100.0% in the diagnosis of BA among infants less than 30 days [29]. This finding was of great value for the early diagnosis of BA.

Specially, the TC thickness may be inconspicuous in the early stages of BA [25,30]. It may be necessary to undergo US examination again during the dynamic course of disease development [22]. In addition, a more objective method of measuring the thickness of TC needs to be further explored.

### 2.3. Porta Hepatis Macro- or Microcyst

**Figure 3:**
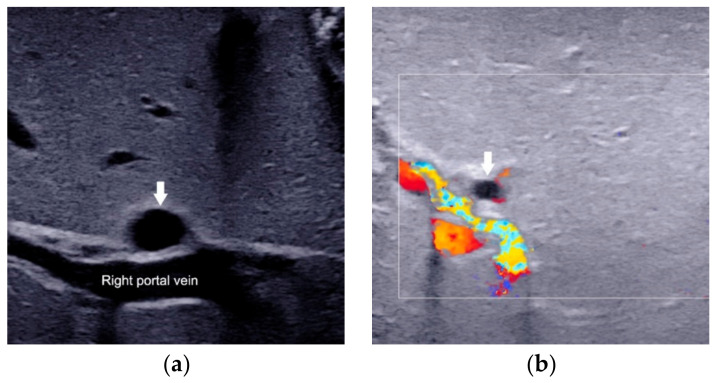
Porta hepatis macrocyst (**a**) and microcyst (**b**) in infants with biliary atresia.

The presence of the porta hepatis macrocyst (Figure 3a) or microcyst (Figure 3b) is a specific sign for BA [31,32]. Macrocyst was defined as the cyst with a diameter >5 mm located in the hepatic pedicle [31,32], while microcyst was defined as the cystv ≤5 mm in diameter in front of the right portal vein at the hepatic portal [31]. Color Doppler can help distinguish cysts from blood vessels. When the porta hepatis cyst is detected in infants with conjugated hyperbilirubinemia on US scan, BA should be highly suspected. In addition, most of BA with microcyst is type III BA, while some of BA with macrocyst is type I BA [33].

Although high specificity (98–99%) was reported in previous study, it only exists in a few infants with BA (sensitivity is only 25%) [31]. Therefore, for infants with conjugated hyperbilirubinemia, even porta hepatis cyst is absent, BA cannot be ruled out.

### 2.4. Enlarged Hepatic Hilar Lymph Node (LN)

**Figure 4:**
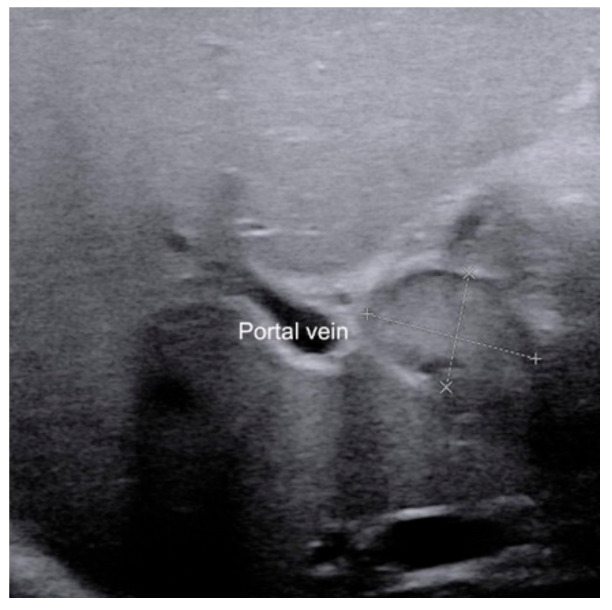
The presence of enlarged hepatic hilar lymph node (calipers) in infants with biliary atresia.

The presence of enlarged hepatic hilar LN, which was defined as the LN located at the porta hepatis and around the hepatoduodenal ligament (Figure 4), is also useful for the diagnosis of BA [28]. The optimal cutoff value was 6.0 mm for the length of LN in the identification of BA, which yielded a sensitivity and specificity of 91.1% and 82.9%, respectively. Combined hepatic hilar LN with gallbladder classification and TC thickness, the sensitivity could even reach 100%. However, the value of hepatic hilar LN was tested in only a single institution. A validation in independent population by other investigators is necessary.

### 2.5. Other Helpful US Features

**Figure 5:**
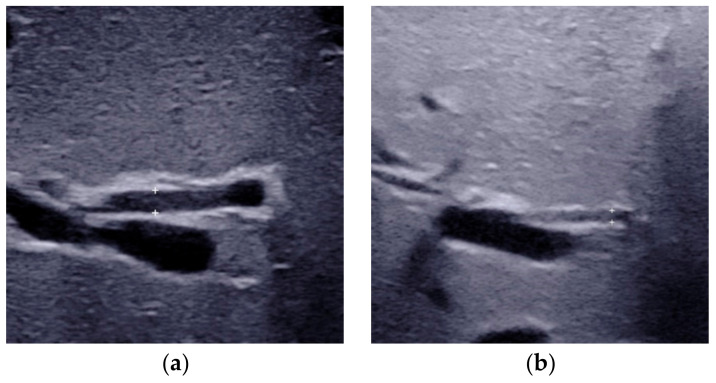
Hepatic artery measurement images of infants with (**a**) and without biliary atresia (**b**).

Several studies have shown that the diameter of HA in infants with BA (Figure 5a) is larger than that of non-BA (Figure 5b) [20,34,35,36,37]. Woo et al. [34] proposed an optimal cutoff value of 1.5 mm for HA diameter in the diagnosis of BA, which yielded a sensitivity of 92% and specificity of 87%. However, the mean diameter of HA between BA and non-BA groups varies greatly in different studies [20,34,35,36,37]. According to reports, the mean HA diameter of BA was between 2.1 mm to 2.5 mm, while the diameter of non-BA was between 1.5 mm to 1.9 mm [20,35,36,37]. Obviously, 1.5 mm is not an optimal cutoff value in the diagnosis of BA. Furthermore, the measurement of the diameter of HA is hard to standardize. Due to the unsatisfactory consistency of diameter of HA in different studies, it is not yet a reliable diagnostic indicator in predicting BA.

**Figure 6:**
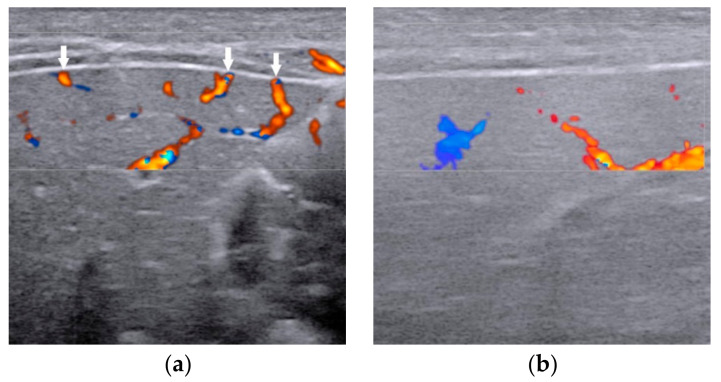
The presence of hepatic subcapsular flow (arrows) in biliary atresia (**a**) and the absence of hepatic subcapsular flow in non-biliary atresia (**b**).

The presence of hepatic subcapsular flow (Figure 6) is a sign of hyperplastic and hypertrophic changes in branches of the hepatic artery, with the sensitivity ranged from 96.3% to 100% and the specificity ranged from 86% to 96.7% in predicting BA [35,36,37]. However, different US equipment and parameter settings make the detection of hepatic subcapsular flow operator-dependent. There is still a lack of clear quantification for comparison between patients.

Since these two indicators were explored in only a few studies. They usually were considered as supportive indicators for the diagnosis of BA.

The various conventional US features used to diagnose BA are summarized in Table 1. As early surgical intervention is critical to the prognosis in patients with BA, the diagnostic sensitivity should be kept at a higher level. Therefore, as long as either the gallbladder or the TC sign is positive, the infant needs to receive surgical exploration or intraoperative cholangiography for further evaluation. If both the gallbladder and TC sign are negative, the presence of porta hepatis cyst can also lead to the diagnosis of BA. Other US features, which can only provide indirect evidence of the onset of BA, are not recommended for diagnosis alone.

## 3. Elastography

Since the progression of liver fibrosis in patients with BA is faster than that in other neonatal cholestasis, the measurement of liver stiffness might be useful to identify BA among infants with conjugated hyperbilirubinemia, especially when all conventional US features are negative. Elastography can be performed to quantify liver stiffness and fibrosis in infants with cholestasis [38,39,40] and to facilitate the differential diagnosis of BA [27,41,42,43,44,45,46,47,48,49,50]. With the development of elastography technology, various types of elastography have been reported for the diagnosis of BA.

Virtual Touch Quantification (VTQ) and Virtual Touch IQ (VTIQ) from Siemens are the first two technologies used for the diagnosis of BA [47], both of which are acoustic radiation force impulse (ARFI)-generated quantitative techniques. As reported, VTQ yielded a sensitivity ranged from 76.9% to 90.9% and specificity ranged from 68.4% to 78.6%, while the sensitivity ranged from 92.3% to 95.5% and specificity ranged from 78.6% to 78.9% of VTIQ [47,48,49,50]. However, limited number of cases in relevant researches make the reliability of VTQ and VTIQ questioned.

Transient elastography (TE) is another type of quantitative elastography technology used to diagnose BA. A cut-off value of 7.7 kPa of TE yielded a sensitivity of 80% and specificity of 97% in infants younger than 90 days [45]. For the infants aged 91 to 180 days, a higher cutoff value of 8.8 kPa could yield higher diagnostic sensitivity (100%) and specificity (100%) [51]. However, the inability to choose different locations for the region of interest limits the clinical applicability of TE [52] in children.

**Figure 7:**
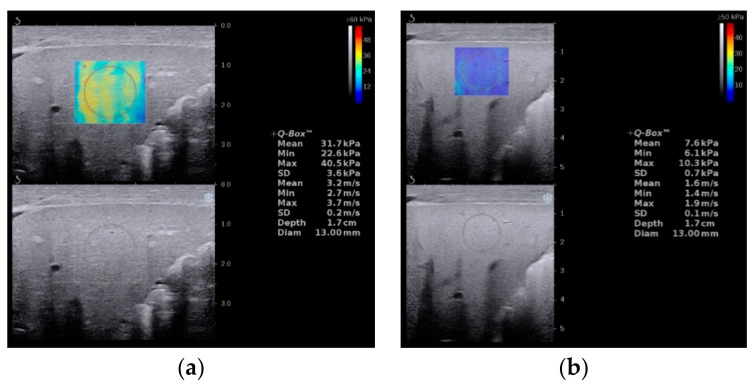
Liver stiffness measurement images of biliary atresia (**a**) and non-biliary atresia (**b**).

Supersonic shear wave elastography (SSWE) is a recently developed elastography system based on high frame-rate shear wave technology. It is based on capturing shear wave speed propagation, which presents a map of the elasticity in one area and allows stiffness quantitative analysis [27,41,47]. In our previous study, the cutoff value of SSWE for differentiating BA (Figure 7a) from non-BA (Figure 7b) was determined to be ≥10.2 kPa, with AUC 0.790, sensitivity 81.4% and specificity 66.7% [27]. Other SSWE thresholds, such as 8.86 kPa proposed by Wang et al. [41] and 7.10 kPa proposed by Liu et al. [42], yielded AUC 0.997 and 0.82, respectively. Fluctuations in AUC values may be caused by differences in age and serum biochemical index levels between different cohorts.

Although elastography has shown good diagnostic capabilities for BA, its performance was inferior to that of conventional US [27,43,53]. The increment of liver stiffness value in patients with BA is the result of intrahepatic cholestasis. Other types of intrahepatic cholestasis disease can also cause liver fibrosis and consequently raise the liver stiffness. Therefore, elastography is more suitable to be considered complementary to conventional US than for making diagnosis alone. Recently, a diagnostic nomogram incorporating the SSWE, gallbladder abnormalities and age showed good discrimination in diagnosing BA, with AUC of 0.898 [54]. Chen et al. [55]. proposed an algorithm with risk stratification to distinguish BA, which was composed of five predictors: shear wave speed, TC sign, GGT, gallbladder abnormalities and clay stool. This algorithm showed promising results with an AUC of 0.983. These findings indicate that the combination of elastography, US features and clinical data can obtain a better predictive value for the diagnosis of BA than that yielded by using elastography alone [54,55]. At present, the greatest clinical value of elastography is to assess the liver fibrosis stages, so as to predict the prognosis after KPE [46,56,57]. For the diagnosis of BA, elastography is more suitable as an aid to conventional US, especially when conventional US alone does not provide a definite diagnosis.

## 4. US-Guided Percutaneous Cholecystocholangiography with Microbubbles

For those infants with a persistent rise of direct bilirubin level but equivocal US results, US-guided percutaneous cholecystocholangiography (PCC) may be used as another less invasive alternative to laparoscopic cholangiography if their gallbladder is full [58,59,60]. As previously reported, the diagnostic performance of US-guided PCC was better than that of conventional US [58,59]. Furthermore, it was helpful to be used for preoperatively differentiating subtypes of BA.

Infants who meet the following indications are recommended to receive US-guided PCC for further evaluation: (1) serum total bilirubin level >31.2 mmol/L; (2) with type IV gallbladder and (3) with negative or equivocal positive TC thickness (0 mm–2.3 mm) [58]. However, infants who have the following conditions should not receive the procedure: (1) intolerant to general anesthesia; (2) with invisible gallbladder lumen; (3) with coagulation dysfunction and (4) with a serious infectious disease.

**Figure 8:**
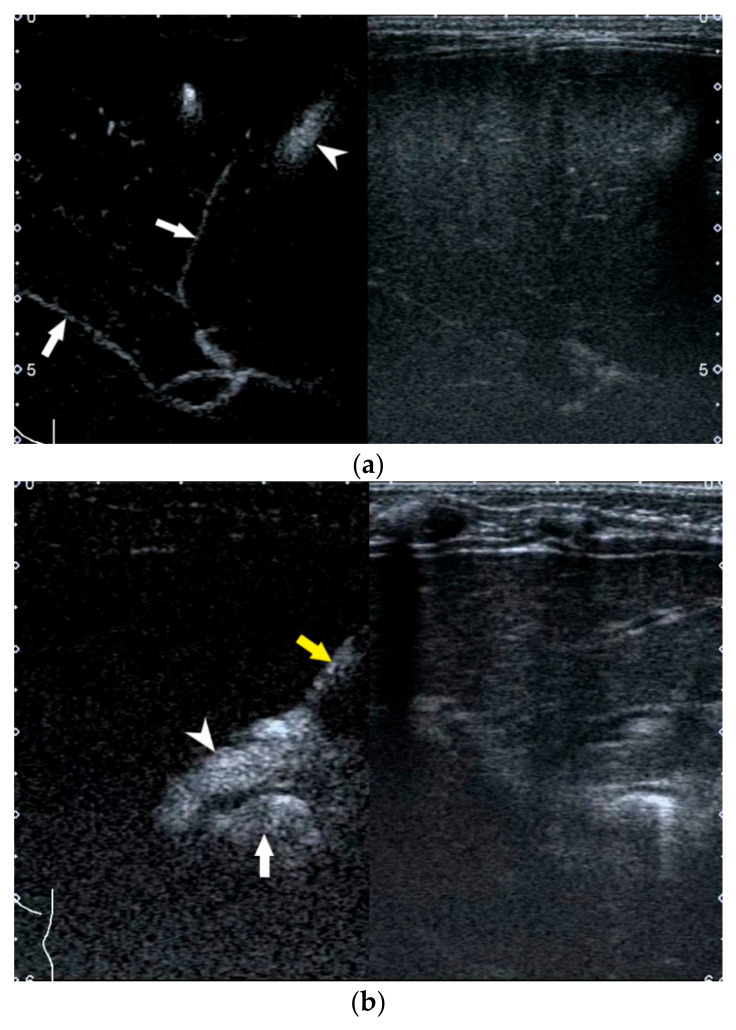
Image obtained at US-guided percutaneous cholecystocholangiography with microbubbles. (**a**) Gallbladder is filled with contrast material (arrowhead) and contrast material flows into intrahepatic bile ducts (arrows) in an infant without BA. (**b**) Contrast material flows along the puncture needle (yellow arrow) into the gallbladder (white arrowhead) and then into bowel (white arrow) in an infant with BA. No contrast material flows into the intrahepatic bile duct.

Infants should be fasted for more than 4 h and then conventional US should be performed before US-guided PCC to ascertain the presence of a filled gallbladder that is potentially available for puncturing. Afterwards, general anesthesia is induced and US-guided PCC is performed with a linear transducer. After percutaneous puncture through the anterior wall of the gallbladder into the gallbladder lumen, the diluted microbubble contrast agent is injected via the puncture needle to allow observation of the distribution of the contrast agent in the biliary system. BA is excluded if the gallbladder, common hepatic duct, common bile duct, and bowel are seen to fill with microbubble (Figure 8a). BA is confirmed if the common hepatic duct is invisible (Figure 8b).

Although US-guided PCC has only been performed in a few cases at present, it seems that serious complications of this procedure are rare [58,59]. However, it is currently only applicable to infants with a well-filled gallbladder (length > 1.5 cm). Further studies are necessary to evaluate the applicability and limitations of the procedure.

## 5. Artificial Intelligence Based on US Gallbladder Images

AI, powered by advances in computation and immense amounts of datasets, has been shown superior or comparable to human experts in many US data analysis tasks, such the diagnosis of thyroid nodules [61,62], breast cancer [63,64], congenital heart disease [65] and fetal brain abnormalities [66]. Driven by the recent advances in deep learning technologies, AI may have the potential to revolutionize BA diagnosis from US images particularly in rural area without relevant expertise.

Recently, an ensemble deep learning model from US gallbladder images has been developed, which adopted two types of effective AI techniques called deep convolutional neural networks (CNNs) and ensemble learning [67]. In this study, the training cohort was randomly separated into five complementary subsets, four of which were used each time to train a CNN, and the remaining subset was used for validation. Thus, five CNNs were trained with the training cohort and then the output predictions of these CNNs were averaged to predict the diagnosis of each test image, resulting in an ensemble deep learning model.

The ensemble deep learning model yielded a sensitivity 93.1% and specificity 93.9% on the external validation dataset, superior to that of three human experts [61]. With the help of the model, the performances of human experts with various levels were improved. It proves that AI can provide a solution to help radiologists improve their diagnosis of BA, particularly for the junior radiologists without relevant expertise.

In order to avoid the inconvenience of copying US images to a computer during testing, a smartphone app was developed to test the gallbladder photograph taking by the smartphone. The image quality of the photograph would be inevitably affected by this imaging process, which requires the radiologist to retain the original image information as much as possible during picturing (e.g., by making camera viewing direction perpendicular to the machine screen). The diagnosis based on smartphone photos of US gallbladder images through a smartphone app still yielded expert-level performance, which proves that the smartphone app can assist the radiologists in practice.

This ensembled deep learning model is the first attempt of AI based on US images in the BA field. Compared with radiologists, AI may be able to more clearly and objectively identify some ambiguous US features. Thus, the automatically detection of other US features by AI is needed as part of future work to help further improve the diagnosis of BA. It is worth trying that BA can be diagnosed through AI automatic detection and measurement of TC thickness, or through AI to extract more features from elastography images, not just through a single liver stiffness measurement value.

## 6. Summary

**Figure 9:**
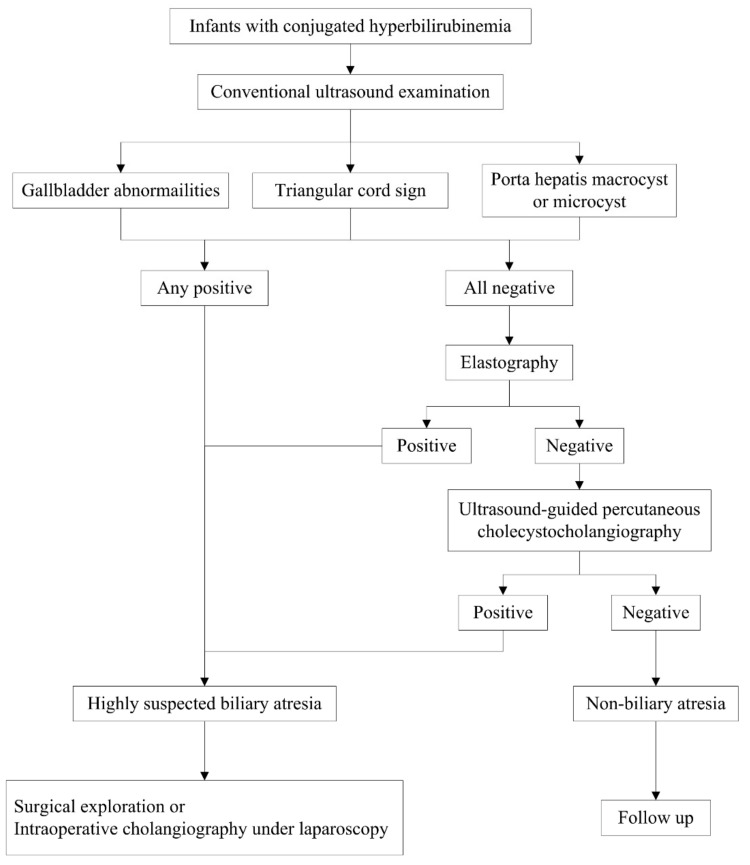
Diagnostic flowchart of biliary atresia.

Since BA can lead to poor outcomes without promptly recognition and treatment, infants with a prolonged jaundice over 2 weeks must always be investigated to rule out BA. US plays a critical role in the working up or ruling out BA among infants with conjugated hyperbilirubinemia (Figure 9). Gallbladder abnormalities, TC sign and porta hepatic cyst(s) are the direct features reflecting the abnormalities of biliary system in infants suspected of BA. Thus, when infants with conjugated hyperbilirubinemia present one of these features on US, BA should be highly suspected and surgical exploring should be recommended. If the infants present no positive conventional US findings, elastography and US-guided PCC can be recommended for further evaluation. MRCP and hepatobiliary scintigraphy offer limited help for the diagnosis of BA with the short of low specificity [68]. Only in some cases with equivocal US diagnosis, additional MRCP and hepatobiliary scintigraphy may provide valuable information about the normal patency of the extrahepatic biliary tree and can reduce unnecessary laparotomy [68,69].

With the help of new technologies, such as elastography US, contrast enhanced US and AI techniques, the diagnostic performance of BA is significantly improved. However, the timely diagnosis of BA among infants with conjugated hyperbilirubinemia is still a challenge. In the future, newly developed AI algorithm based on multimodal US features (conventional US and elastography US) combined with clinical data (such as serum GGT level and age) might provide a solution for this challenge.

## Figures and Tables

**Table 1:** Summary of various conventional ultrasound features used for diagnosing biliary atresia.

US Features	Definition	Positive	Negative	Diagnostic Value of BA when Positive
Gallbladder abnormalities	Abnormalities in the length of the gallbladder lumen, the integrity of the mucosal lining, and the degree of contraction of the gallbladder after feeding.	① The length of the fully filled gallbladder lumen <1.5 cm; ② Lack of smooth and complete echogenic mucosal lining; ③ No contraction of the gallbladder after feeding	① The length of the fully filled gallbladder lumen >1.5 cm with smooth and complete hyperechogenic mucosal lining; ② Incompletely filled lumen of the unfilled gallbladder with smooth and complete hyperechogenic mucosal lining	Strongly suggest BA
Triangular cord sign	The thickness of the echogenic anterior wall of the right portal vein, with or without HA.	>2.0 mm not including HA, or >4.0 mm including HA	≤2.0 mm not including HA, or ≤4.0 mm including HA	Strongly suggest BA
Porta hepatis macro- or microcyst	The cyst in front of the right portal vein at the hepatic portal.	Presence	Absence	Strongly suggest BA
Hepatic hilar lymph node	The lymph node located at the porta hepatis, around the hepatoduodenal ligament.	Presence	Absence	Possible BA
HA diameter	Measured at the level of right proximal HA running parallel to the right portal vein.	2.1 mm to 2.5 mm	1.5 mm to 1.9 mm	Not recommended for diagnosis alone
Hepatic subcapsular flow	Vascular structures continued to the liver capsular surface on color Doppler US images.	Presence	Absence	Not recommended for diagnosis alone

Note: US, ultrasound; BA, biliary atresia; HA, hepatic artery.

## Data Availability

Not applicable.
